# Catnip Oil, Monoterpenes Carvacrol, and Citronellol as an Effective Repellent Against *Cimex lectularius* (Hemiptera: Cimicidae)

**DOI:** 10.3390/insects17070705

**Published:** 2026-07-07

**Authors:** Souvic Sarker, Jin-Jia Yu, Qingli Wu, James E. Simon, Changlu Wang

**Affiliations:** 1 Department of Entomology, Rutgers, The State University of New Jersey, New Brunswick, NJ 08901, USA; souvicsarker08@gmail.com (S.S.); jy767@sebs.rutgers.edu (J.-J.Y.); 2 Department of Plant Biology, Rutgers, The State University of New Jersey, New Brunswick, NJ 08901, USA; qlwu@sebs.rutgers.edu (Q.W.); jimsimon@sebs.rutgers.edu (J.E.S.)

**Keywords:** catnip oil, nepetalactone, DEET, repellency, bed bugs, carvacrol, citronellol

## Abstract

The common bed bug (*Cimex lectularius* L.) is an obligate blood-feeding insect that spreads easily through human activity. Catnip (*Nepeta cataria* L.) oil has emerged as a promising natural repellent for bed bugs, but previous studies have been limited to laboratory conditions. In this study, we assessed the effectiveness of a catnip-based formulation (20% catnip CCC oil: 10% catnip oil cultivar CR9, 5% carvacrol, 5% citronellol) applied to fabric against bed bugs using simulated field tests, a human finger stimulus test, and field trials. *N*-Diethyl-meta-toluamide (DEET) was included as a benchmark in the simulated field test. In these tests, both 20% catnip CCC oil and 20% DEET achieved >94% repellency within 24 h, with comparable performance after 2–3 d of aging. After 5 d, 20% catnip CCC oil showed significantly reduced repellency compared to DEET. In the human finger stimulus test, 20% catnip CCC oil remained effective for up to 6 d, whereas in infested apartments, significant repellency lasted only one day. These findings suggest that 20% catnip CCC oil applied to fabric can provide short-term protection against bed bug introductions under natural conditions. Future work should explore optimized barrier dimensions and slow-release formulations to extend efficacy.

## Simple Summary

Bed bugs (*Cimex lectularius* L.) remain persistent urban pests, and few effective repellent tools are available for preventing their spread. Catnip oil has shown strong repellency in laboratory studies, but its performance under realistic conditions is less understood. We evaluated a fabric-applied formulation containing catnip oil, carvacrol, and citronellol (20% catnip CCC oil) using simulated field tests, a human finger stimulus assay, and field trials in infested apartments. The formulation showed repellency comparable to 20% DEET for up to three days in semi-field arenas and remained effective for up to six days in the human-stimulus test. However, in naturally infested apartments, repellency lasted only one day. These results indicate that catnip CCC oil can provide short-term protection against bed bug introductions and may serve as a complementary tool within integrated pest management programs.

## 1. Introduction

The common bed bug, *Cimex lectularius* L. (Hemiptera: Cimicidae), is a challenging pest to manage due to its cryptic behavior, resistance to insecticides, and the limited number of effective chemical options for bed bug control [1,2,3]. As an obligate blood-feeding insect, bites from bed bugs cause itching, rashes, and pain [4,5,6], and may occasionally induce systemic reactions [7,8] and mental health effects such as anxiety, stress, and sleeplessness [9]. Infestations are common in multi-unit housing due to close proximity and active dispersal between units [10,11]. Bed bugs also spread passively by human transport while harboring on clothing, luggage, wheelchairs, shoes, and other belongings, creating risks in homes, hotels, healthcare facilities, theaters, and public transport [12]. Visitors may acquire bed bugs even during short stays (15 min to a few hours), as hungry bed bugs actively seek hosts using heat, carbon dioxide, and odor cues [13,14,15].

While eradication remains the only long-term solution, there is interest in temporary measures to reduce incidental contact during short-term exposure, such as during inspections or travel. One proposed approach is applying repellent materials to non-skin surfaces (e.g., clothing or luggage) to deter bed bugs from crossing the repellent residue. However, repellents are not considered primary bed bug management tools and cannot replace proven IPM tactics such as heat treatments, desiccant dusts, or residual insecticides [10,16]. Our study focused on this narrow use case: repellent-treated fabric for short-term deterrence, not on topical application to human skin.

Since the 1950s, DEET (*N*, *N*-diethyl-m-toluamide) has been the most widely used insect repellent [17]. DEET is effective against many blood-feeding arthropods, including mosquitoes, ticks, and biting flies [18,19,20], and laboratory studies show DEET can repel bed bugs for several hours [21,22]. However, DEET is not registered for bed bug use in the U.S., and its odor, skin discomfort, and potential side effects limit acceptability [23,24]. These factors have driven interest in plant-based repellents, which are perceived as “natural” but often may be less effective than DEET and can also cause dermatological reactions [25,26].

Catnip oil (*Nepeta cataria* L.), rich in nepetalactone, has shown strong short-term repellency against bed bugs in laboratory assays [27]. At 25%, catnip oil from cultivar ‘CR9’ achieved 100% repellency within 24 h in laboratory assays, which was higher than the 92% repellency observed for DEET at the same concentration, though its efficacy declined after aging due to volatility [27]. Catnip oil has also been reported as a mosquito repellent [28,29]. Toxicity tests suggest catnip oil is relatively safe for non-topical use but may cause mild irritation [26]. Importantly, catnip oil has a strong odor, which may limit practical adoption and requires transparent discussion.

Despite promising lab results, there is limited information on catnip oil performance under realistic conditions or when human stimuli are present. Repellent effectiveness can be influenced by environmental factors and competing host cues [30]. Therefore, further research is needed to evaluate catnip-based repellents in scenarios that mimic real-world exposure. Catnip oil loses repellency quickly due to its high volatility [27], so a formulation was developed in which catnip oil is supplemented with two additional terpenes, carvacrol and citronellol, which have demonstrated repellent activity and may help improve residual performance [31]. This study investigates the repellency of a formulated 20% catnip CCC oil (10% CR9 catnip oil + 5% carvacrol + 5% citronellol) [31] applied to fabric substrates intended for garments or luggage. Specifically, the objectives were to (1) compare its repellency to 20% DEET under simulated field conditions, (2) assess repellency in the presence of human stimulus, and (3) evaluate performance in naturally infested apartments. We emphasize that this work does not propose repellents as standalone solutions. Instead, it explores their potential as complementary, short-term measures within IPM.

## 2. Materials and Methods

### 2.1. Bed Bugs

Four field-collected *C. lectularius* populations (Irvington, Irvington 624-5G, Masiello, and Cotton) were used in this study. The bed bugs were collected from apartments in New Jersey, USA, between 2012 and 2018. They were maintained in plastic containers (4.7 cm height and 4.7 cm diameter) and fed every one to three weeks with defibrinated rabbit blood (Hemostat Laboratories, Dixon, CA, USA) using the Hemotek membrane-feeding system (Discovery Workshops, Accrington, UK). The bed bug containers were kept in an environment chamber at 25 °C, 45 ± 10% RH, and a 12:12 h (L:D) photoperiod. Males or third to fifth instar nymphs were fed 14–24 d prior to all assays. Only adult males and nymphs were used in the experiment to avoid the risk of egg-laying and potential infestation by females. Previous studies have shown that male and female *C. lectularius* exhibit similar avoidance behaviors toward plant-derived repellents [30]; therefore, it was assumed that females would have similar responses to the tested repellents as males.

### 2.2. Essential Oil Preparation and Analysis of Chemicals

#### 2.2.1. Chemicals

Essential oil from the catnip cultivar CR9 was prepared using the method described by Patel et al. [32]. Briefly, catnip plants were grown under field conditions at the Rutgers Agricultural Experiment Station in Pittstown, New Jersey. The plants were dried and hydrodistilled using a Clevenger-type apparatus [33]. The essential oil was collected and separated from the hydrosol. The essential oil was dried over anhydrous sodium sulfate and analyzed by gas chromatography/mass spectrometry (GC/MS) using the Shimadzu Gas Chromatograph 2010 Plus coupled with a TQ8040 Mass Spectrometer (Shimadzu Scientific Instruments, Kyoto, Japan). The individual terpenes citronellol and carvacrol were obtained from Sigma Aldrich (St. Louis, MO, USA). DEET (97%), isopropanol (100%), 15% polyethylene glycol-200 (PEG-200), and 0.05% butylated hydroxytoluene (BHT) were obtained from Fisher Scientific (Hampton, NH, USA).

#### 2.2.2. Preparation of Catnip Essential Oil and Terpene Mixture

A mixture product consisting of citronellol, carvacrol, and catnip oil (hereafter referred to as 20% catnip CCC oil) from cultivar CR9 is designed to provide longer-term repellency than catnip oil alone. Citronellol has a history of showing some repellency, but it is not as effective as carvacrol, the main aromatic volatile in the essential oil of oregano, which exhibits strong repellency against bed bugs [30]. The 20% (*w*/*v*) catnip CCC oil was prepared by adding 10% catnip cultivar CR9 essential oil, 5% carvacrol, and 5% citronellol to a solution of isopropanol containing 15% PEG-200 and 0.05% BHT. A 20% DEET solution was also prepared by diluting DEET in isopropanol containing 15% PEG-200 and 0.05% BHT.

#### 2.2.3. Analysis of the Catnip Repellent Products via Gas Chromatography and Mass Spectrometry (GC-MS)

A Restek SH-Rxi-5Sil MS 30 m × 250 mm × 0.25 μm column (Restek Co., Bellefonte, PA, USA) was used to separate the compounds following the injection of a 1 μL aliquot of the samples. The injection temperature was 250 °C. Helium was used as the carrier gas, and the flow rate was set to 1 mL/min. The temperature of the column oven was set to and maintained at 35 °C for a duration of 4 min. The temperature was then raised to 250 °C at a rate of 20 °C/min and held at 250 °C for 1.25 min. For the MS conditions, the interface temperature was 250 °C and the ion source temperature was 200 °C, with the detector voltage set at 0.04 kV and a threshold of 1000. A range of 45 *m*/*z* to 500 *m*/*z* was used to scan the fragment ions, and the mass spectra were used for compound identification. The mass spectra of the compounds were compared to those in the NIST05s.LIB, NIST05.LIB, W10N14.lib, and W10N14R.lib. mass spectral libraries. Furthermore, the compounds’ identities were confirmed by a series of n-alkanes (C8–C18) used to generate Kovats indices as well as by the literature [34].

### 2.3. Testing Repellency of 20% Catnip CCC Oil Under Semi-Field Conditions

**Figure 1:**
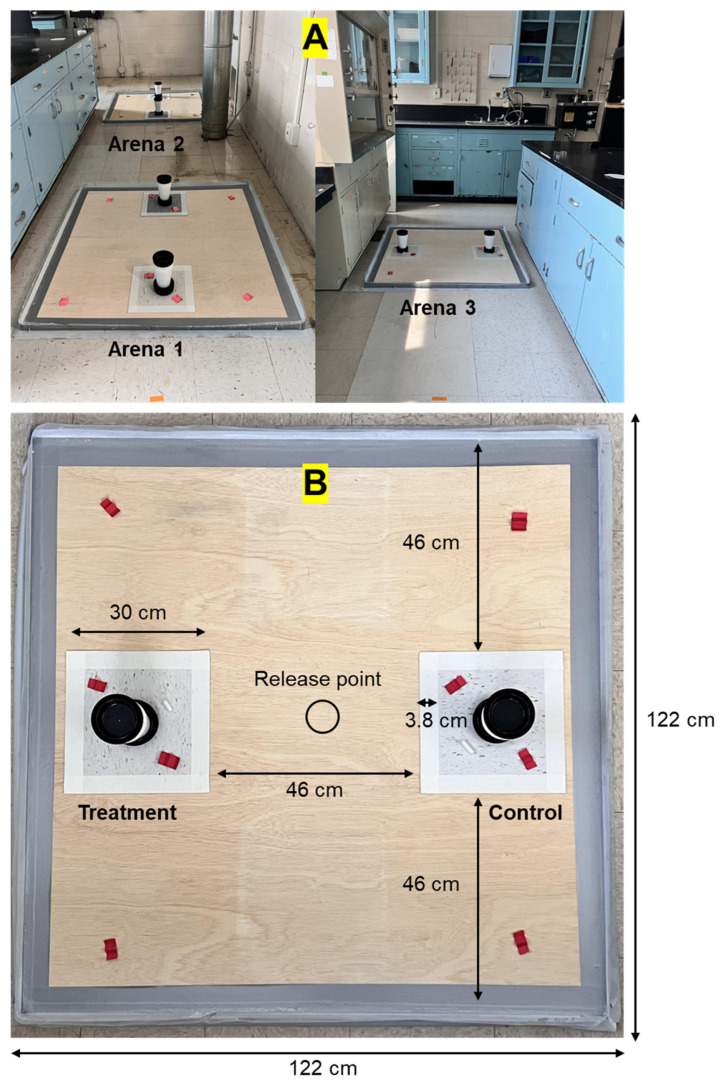
Experimental setup comparing the repellency of catnip CCC oil and DEET against field populations of *Cimex lectularius*. (**A**) Arenas in a room; (**B**) an arena showing a linoleum tile treated with 20% catnip CCC oil solution and another tile treated with isopropanol + PEG as a control. Depending on the test, the two tiles could also be 20% DEET vs. control or 20% catnip CCC oil vs. 20% DEET.

This experiment was conducted in three rooms, each containing three plywood arenas (122 × 122 cm) (Figure 1A,B). In each arena, two linoleum tiles (30 × 30 cm) were placed. Each tile had a 3.8 cm wide sports tape (CVS Health™, Woonsocket, RI, USA) attached to all four borders, leaving a 523 cm^2^ untaped area in the center. Treatments were applied only to the sports tape, not to the tile surface. The treatment combinations were as follows: one tile with tape treated with 20% catnip CCC oil and the other with tape treated with isopropanol + PEG (control); one tile with tape treated with 20% DEET and the other with tape treated with isopropanol + PEG (control); or one tile with tape treated with 20% catnip CCC oil and the other with tape treated with 20% DEET. Each tape was treated with 5 mL of the respective solution, corresponding to 69.6 µg of active ingredient per cm^2^. This amount is calculated as 1000 mg total actives in a 5 mL solution divided by 1436 cm^2^ of tape surface, yielding an application rate of 69.6 µg cm^−2^. This value is based on gravimetrically measured mass, independent of constituent densities.

The two linoleum tiles were placed along two opposite sides of each arena, and the distance separating each linoleum tile from the bed bug release point measured 20 cm. A total of 100 bed bugs comprising 50 nymphs (3rd to 5th instars) and 50 adult males were released at the center of the arena and confined within a plastic ring (9 cm in diameter and 4 cm in height) for 24 h to allow acclimation before the assay began. Inside the ring, a folded piece of red cardboard (3 × 2 cm) was provided as a harborage for the bed bugs. After placing the linoleum tiles in the arena, a pitfall trap (Black Climbup HD-9.8 cm in diameter and 2.0 cm in height) (Susan McKnight Inc., Memphis, TN, USA) with an insulated thermos water bottle containing approximately 250 g of dry ice, one 1.5 mL microcentrifuge tube containing 400 µL of bed bug lure [35], and two folded pieces of paper for harborage were placed on top of each linoleum tile. The tube had a 1 mm diameter hole drilled in the cap to release the lure. From our preliminary experiment, 250 g of dry ice can last at least 9 h. Additional dry ice was added at 8 h and 16 h to provide a continuous supply of CO_2_ during the bioassay. A thin layer of Insect-a-Slip (PTFE Fluoropolymer Dispersion, BioQuip Products, Rancho Dominguez, CA, USA) was applied to the interior surface of the interceptors to prevent bed bugs from escaping once captured. The plastic ring that contained the bed bugs was immediately removed after the CO_2_ and lure were introduced. Four folded papers were placed at each corner of the plywood arena to provide resting sites for bed bugs that did not choose either of the treated tiles. This design mimics real-world conditions where bed bugs have multiple harborages available and prevents stress or unnatural clustering during the extended observation period. Additionally, since a dry ice trap was used on each tile as a CO_2_ source to attract bed bugs, the folded papers ensured that individuals could avoid treated areas if they were repelled, maintaining a more realistic behavioral choice scenario. Because each tile contained strong attractants (dry ice as a CO_2_ source and a chemical lure), preliminary trials showed that bed bugs that avoided crossing a treated band frequently accumulated along arena edges or attempted to climb walls. This created stress-induced, non-natural behavior and reduced the number of insects making meaningful contact with the treated or control surfaces. The corner harborages acted only as neutral resting sites to prevent this artifact. They were placed deliberately far from both tiles and did not function as alternative choice points. Bed bugs that entered these harborages were excluded from repellency calculations. Duct tape (Home Depot, Milltown, NJ, USA) was used to encircle the plywood edges, preventing bed bugs from escaping.

In the aging test, 5 mL of each solution was dispensed directly onto the sports tape using a micropipette, and a cotton ball pre-wetted to saturation with the same solution was used only to spread the liquid evenly across the tape surface, preventing absorption of the applied dose. The treated tapes were then aged for 30 min, 2 d, 3 d, or 5 d before being placed in the arena. The linoleum tiles with treated bands were kept under laboratory conditions of 20–23 °C and natural lighting. The experiment involved three bed bug populations (Irvington 624-5G, Masiello, and Cotton), with each population assigned to a specific experimental room. Each experimental room contained three experimental arenas. They were used to compare 20% catnip CCC oil versus control, 20% DEET versus control, and 20% catnip CCC oil versus 20% DEET, respectively. This setup was repeated twice, resulting in a total of six replications for each treatment combination. Plywood arenas were cleaned with control solution (isopropanol + PEG) after the first replication. After 1, 2, 4, 8, and 24 h of dry ice introduction, the number of bed bugs that crossed the treated bands (either in the interceptor or hiding in folded papers on tiles) was recorded. After the final data collection, all the bed bugs from the arena, interceptor, and folded paper were removed. The positions of the two treatment tiles were switched before running the next test. For each aging period (30 min, 2 d, 3 d, and 5 d), new linoleum tiles with freshly treated tape were prepared and aged separately, and new paper harborages were used for each replicate. No tiles or harborages were reused.

### 2.4. Human Finger Stimulus Test

**Figure 2:**
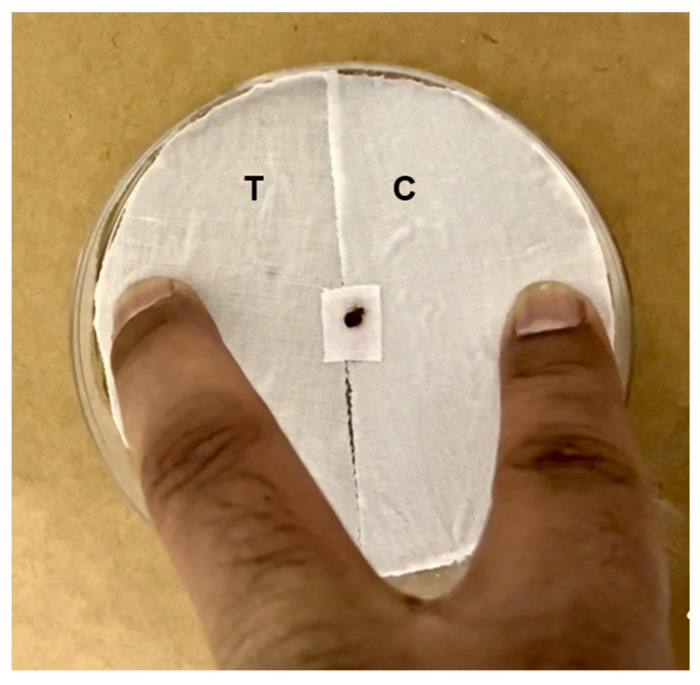
Horizontal choice bioassay where *C. lectularius* are challenged to approach and cross isopropanol + PEG-treated (C) versus 20% catnip CCC oil solution-treated (T) textile surfaces in order to reach a human finger stimulus.

The choice test bioassay was conducted as described by Eisen et al. [36]. Plastic Petri dishes of 100 mm in diameter (Fisher Scientific, Pittston, PA, USA) were used as experimental arenas (Figure 2). Each plastic Petri dish was lined with a textile surface (100% cotton, Waverly Inspirations, Walmart Stores, Inc., Bentonville, AR, USA). The textile surface was split into two equal halves to compare two textile surfaces: one treated with isopropanol + PEG solution (control) and the other with 20% catnip CCC oil solution. The textile was washed with soapy water and dried prior to use to remove potentially harmful chemicals that could be toxic to bed bugs. A total of 0.5 mL of each solution (2.54 mg of catnip CCC oil per cm^2^) was evenly applied to each textile surface using a micropipette, and a cotton ball was used only to spread the solution evenly across the textile surface.

To evaluate the aging effect of 20% catnip CCC oil on bed bug repellency, adult male bed bugs from the Irvington population were used. The experiment was conducted in a walk-in chamber during the light phase. Three Petri dishes were used as experimental arenas. On each day, 15 bed bugs were tested individually in each of the three Petri dishes, releasing each insect one at a time onto the introduction zone located at the center of the arena directly on the line where the treated and control textile pieces met, resulting in 45 individual replicates per day. Human fingers were positioned 10 mm beyond each textile section as a stimulus to guide bed bug movement. Two fingers (index and middle) were used as paired human stimuli. Before each test, the portion of the finger that contacted the fabric was cleaned with the control solution (isopropanol + PEG) to prevent unintended chemical cues. During each set of trials within a Petri dish, the left vs. right positions of the index and middle fingers were alternated after each insect test. In addition, the orientation of the treated vs. control textile halves was reversed when moving to the next Petri dish. These independent rotations ensured that neither finger nor treatment consistently occupied the same side, thereby eliminating positional bias. Each trial lasted for up to 5 min, and the movement of each bed bug toward either the treated or control side was recorded. When a bed bug reached the finger stimulus, the trial was stopped for that individual. Bed bugs that reached the finger did not avoid contacting it, indicating that the finger itself was not repellent and did not influence repellency behavior. The same Petri dishes were reused each day to assess the residual repellency of the treatment. Potential chemical residues from bed bugs were unlikely to affect subsequent tests because the insects made only brief contact with the textile before reaching the finger stimulus, and they were removed immediately. Bed bugs in this assay do not engage in probing, feeding, or prolonged resting on the fabric, minimizing the possibility of depositing chemical cues. Testing continued daily for 7 days, until no significant repellency was observed. After each test, the treated Petri dish was placed on a shelf in the laboratory at 20–23 °C under a natural light cycle.

### 2.5. Testing Repellency of 20% Catnip CCC Oil in Bed Bug-Infested Apartments

**Figure 3:**
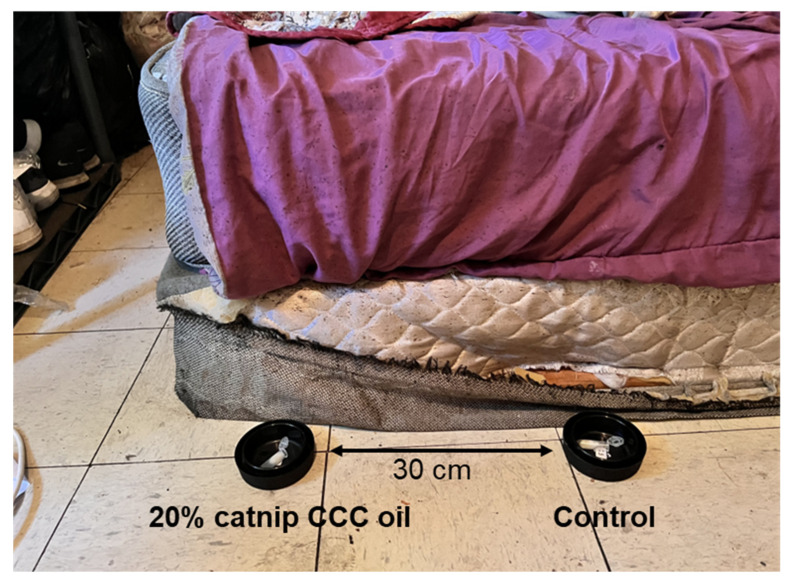
A pair of black Climbup HD interceptors treated with 20% catnip CCC oil or isopropanol + PEG beside an infested mattress in an apartment.

The repellency of catnip CCC oil was further evaluated in seven bed bug-infested apartments in New Jersey, USA. Six apartments from a high-rise apartment building located in Linden city and one apartment from a high-rise apartment building in Paterson city were selected. Black Climbup HD interceptors were used to evaluate the repellency of 20% catnip CCC oil. A pair of interceptors, one treated with a 20% catnip CCC oil solution and another with an isopropanol + PEG solution (control), was used (Figure 3). Each interceptor contained one 1.5 mL microcentrifuge tube containing 400 µL of bed bug lure. The exterior walls of the interceptors had black surgical tape to facilitate bed bugs climbing up the interceptor and entering the trap. An amount of 1 mL of solution (2.65 mg of catnip CCC oil per cm^2^) was evenly applied to the black surgical tape of each interceptor using a cotton ball. Pairs of interceptors were placed under the bed or behind the mattress, under the sofa, under the chair, under the table, and around the perimeter of the room. Interceptors were not placed under furniture legs. The distance between the two interceptors in each pair was approximately 30 cm (Figure 3). A total of 70 pairs of interceptors were placed in seven apartments. Interceptors were checked, and the number of bed bugs was counted daily for 3 d. The positions of the two interceptors were switched after each visit to avoid any location effects.

### 2.6. Statistical Analysis

In the semi-field experiment, the repellency index was determined using the following formula: Repellency index = (C − T)/(C + T) × 100, where C represents the number of bed bugs collected from the control panel and T represents the number of bed bugs from the treated panel (catnip CCC oil or DEET). The number of bed bugs on each of the two treated panels (catnip CCC oil and DEET), the repellency index of 20% catnip CCC oil and 20% DEET, the number of bed bugs that reached the finger by crossing the 20% catnip CCC oil-treated textile versus the control textile, and the number of bed bugs in the 20% catnip CCC oil-treated and control interceptors in the field experiment were analyzed using a paired *t*-test. Among the 70 pairs of interceptors installed in the apartments, only those pairs in which at least one interceptor contained bed bugs were included in the data analysis, while pairs with no bed bugs in either trap were excluded from the analysis. All analyses were conducted using SAS 9.4 software [37].

## 3. Results

### 3.1. Composition of the Catnip CR9 Oil

The composition of catnip cultivar CR9 essential oil was analyzed by GC/MS, and the relative peak percentages were identified (Table 1). The oil primarily consisted of monoterpenes with *Z*, *E*-nepetalactone as the major component (79.8% based on the relative % of total EO). Others included caryophyllene oxide (5.9%) and *β*-caryophyllene (5.3%) along with *E*, *Z*-nepetalactone (3.5%) and α-pinene (3.0%). Overall, 98.7% of the constituents were identified.

### 3.2. Repellency of 20% Catnip CCC Oil Under Semi-Field Conditions

**Figure 4:**
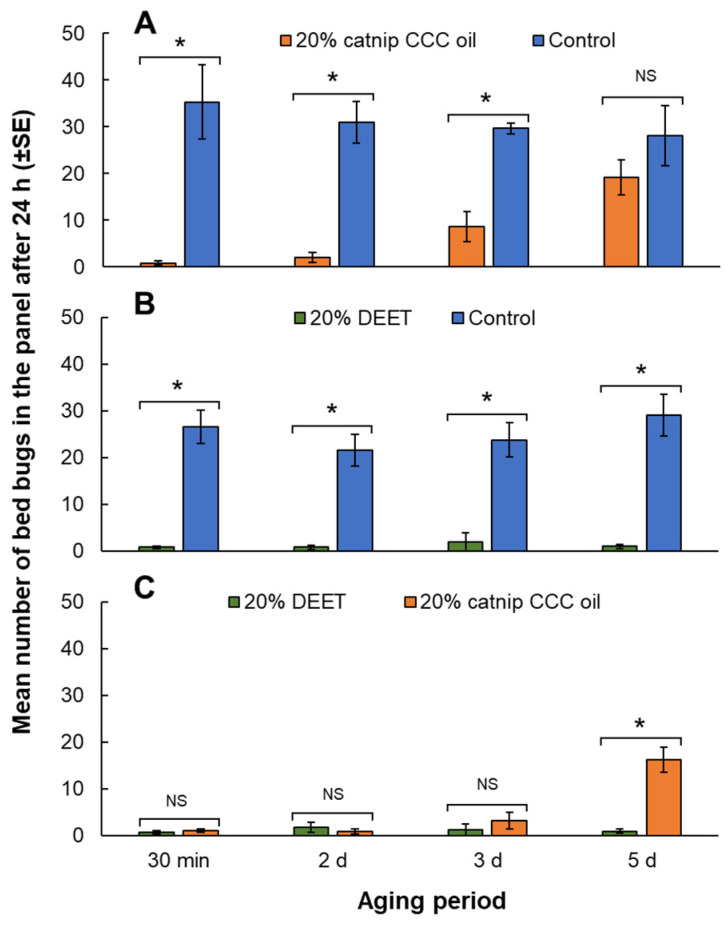
Effect of aging on repellency of two repellents in 122 × 122 cm arenas. (**A**) 20% catnip CCC oil; (**B**) 20% DEET; (**C**) 20% DEET versus 20% catnip CCC oil. * indicates statistical differences between the treatments at each period (paired *t*-test, *p* < 0.05). NS indicates no statistical difference between the two treated panels.

When comparing 20% catnip CCC oil and control, the number of bed bugs that crossed treated bands (either in interceptors or hiding in harborages on tiles) was higher on the control panel than on the repellent treated panel when the aging period was 30 min (*t* = 4.42, *df* = 5, *p* = 0.007), 2 d (*t* = 7.19, *df* = 5, *p* < 0.001), and 3 d (*t* = 6.10, *df* = 5, *p* = 0.002) (Figure 4A). However, after 5 d of aging, the number of bed bugs was similar between the 20% catnip CCC oil-treated panel and the control panel at 24 h after deployment (*t* = 1.00, *df* = 5, *p* = 0.361) (Figure 4A).

In the comparison between 20% DEET and the control, significant repellency was observed for DEET at every aging period (paired *t*-test, *p* < 0.05) (Figure 4B). In the comparison between 20% catnip CCC oil and 20% DEET, the number of bed bugs that crossed the treated bands was similar between the two panels when the aging period was 30 min (*t* = 1.58, *df* = 5, *p* = 0.175), 2 d (*t* = 1.27, *df* = 5, *p* = 0.259) and 3 d (*t* = 2.34, *df* = 5, *p* = 0.067) (Figure 4C). However, after 5 d of aging, the number of bed bugs was higher on the 20% catnip CCC oil-treated panel compared to the DEET-treated panel at 24 h after deployment (*t* = 5.72, *df* = 5, *p* = 0.002) (Figure 4C).

**Figure 5:**
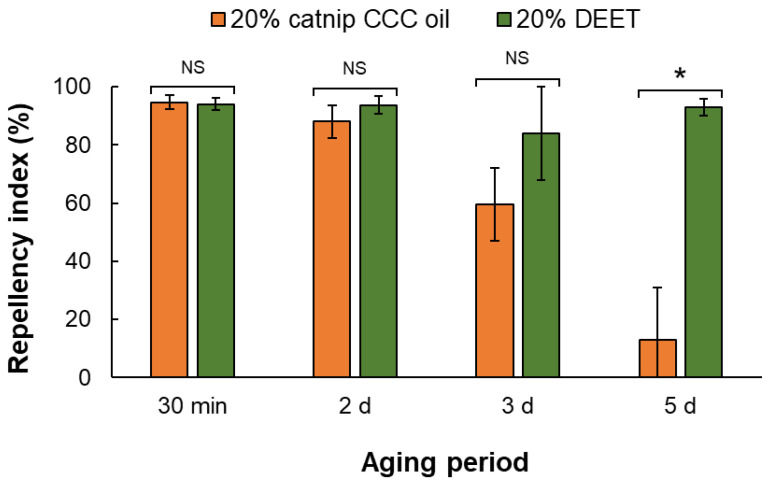
Repellency index of 20% catnip CCC oil and 20% DEET solution in 122 × 122 cm arenas. * indicates statistical differences between 20% catnip CCC oil and 20% DEET solution at each aging period (paired *t*-test, *p* < 0.05). NS indicates no statistical difference between the two treatments.

The mean repellency index of 20% catnip CCC oil and 20% DEET was similar after 30 min, 2 d, and 3 d aging periods (paired *t*-test, *p* > 0.05) (Figure 5). The repellency index of 20% catnip CCC oil was 94.6, 88.0, and 59.4% at 30 min, 2 d, and 3 d aging periods, respectively. However, after 5 d of aging, the mean repellency index of 20% catnip CCC oil reduced to 13 ± 18%, which was significantly lower than 20% DEET (93 ± 3%). Under simulated field conditions, catnip oil exhibited similar repellency to DEET for a minimum of 3 d.

### 3.3. Repellency of 20% Catnip CCC Oil in Human Finger Stimulus Test

**Figure 6:**
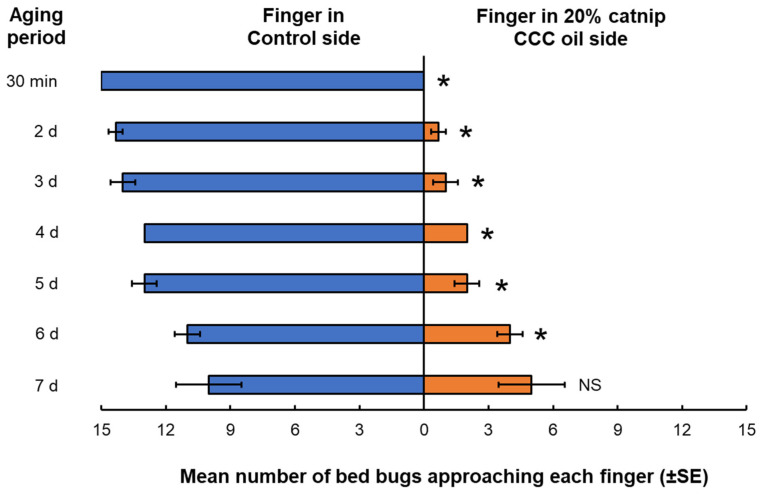
Repellency of 20% catnip CCC oil based on the human finger stimulus test. The Y-axis represents the percentage of adult male bed bugs that reached the finger by crossing the treated or control textile band within a 5 min period. * indicates statistical differences between the treatments at each period (paired *t*-test, *p* < 0.05). NS indicates no statistical difference between the catnip oil-treated textile and the control textile.

Adult bed bug males showed significant avoidance of the catnip oil-treated textile until 7 d of aging periods (30 min: *p* < 0.001, 2 d: *p* = 0.002, 3 d: *p* = 0.008, 4 d: *p* < 0.001, 5 d: *p* = 0.011, and 6 d: *p* = 0.026 (Figure 6). No significant difference in the number of adult male bed bugs that reached the finger by crossing the treated or control textile was found when the aging period reached 7 d (*p* = 0.243). Therefore, in the human finger stimulus test, the catnip CCC oil exhibited a significant repellent effect until 6 d.

### 3.4. Repellency of 20% Catnip CCC Oil in Bed Bug-Infested Apartments

**Figure 7:**
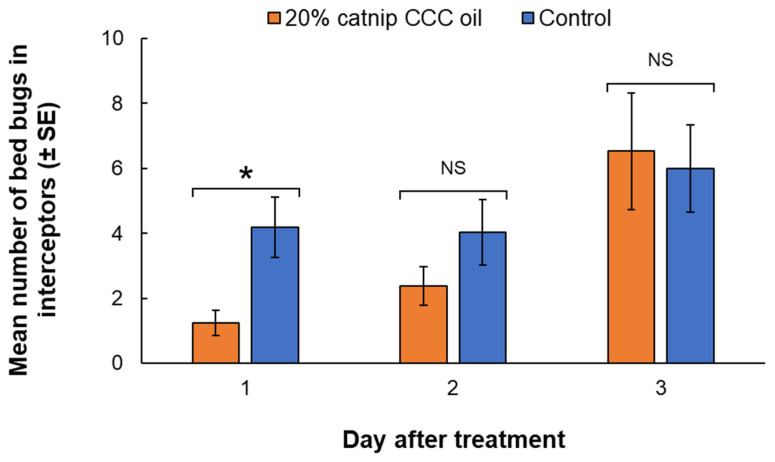
Repellency of 20% catnip CCC oil in bed bug-infested apartments. * indicates statistical differences between 20% catnip oil and control solution (paired *t*-test, *p* < 0.05). NS indicates no statistical differences between 20% catnip oil and the control solution.

A total of 26, 31, and 19 pairs of interceptors had bed bugs caught after 1, 2, and 3 d, respectively. The mean number of bed bugs trapped in 20% catnip CCC oil-treated Climbup was 1.2 ± 0.4, which was significantly lower than that in the control (4.2 ± 0.9) after 1 d of deployment (*t* = 3.74, *df* = 25, *p* < 0.001). No significant differences were found between 20% catnip CCC oil and the control after 2 d and 3 d aging periods (paired *t*-test, *p* > 0.05). Therefore, in apartments, 20% catnip CCC oil showed significant repellency only for 1 d (Figure 7).

## 4. Discussion

This study assessed the effectiveness of catnip CCC oil as a bed bug repellent under laboratory and field conditions using multiple test designs. All experiments demonstrated a significant repellent effect of catnip CCC oil against host-seeking bed bugs, with repellency comparable to DEET for up to 3 days in controlled settings. These findings indicate that catnip CCC oil may serve as a potential alternative to DEET for short-term deterrence.

Previous studies, such as Shi et al. [27], primarily evaluated catnip oil using indirect odor sources (e.g., worn socks) and reported that 25% CR3 and CR9 catnip oils achieved 100% repellency after 24 h, outperforming DEET (92 ± 1%). However, their results also showed that repellency declined substantially after 3 days, and by 28 days, CR9 oil exhibited only 64 ± 4% repellency compared to 92 ± 2% for DEET, while CR3 dropped to 25 ± 3%. To address this limitation, we developed a mixture product (20% catnip CCC oil) combining catnip CR9 essential oil with citronellol and carvacrol, two terpenes with documented repellent properties. Carvacrol, the primary aromatic volatile in oregano oil, has shown strong repellency against bed bugs, while citronellol provides additional deterrent effects [30]. This combination was designed to enhance longevity and broaden the repellent spectrum compared to catnip oil alone. Our findings indicate that while catnip CCC oil maintained repellency comparable to DEET for up to 3 days under semi-field conditions and up to 6 days in human finger stimulus tests, its performance in field trials was limited to 1 day. These results suggest that although the mixture improved short-term efficacy, further optimization, such as slow-release formulations, may be necessary to achieve extended protection in real-world settings.

We selected CR9 essential oil as the primary component of our formulation because previous studies [27] demonstrated that CR9 exhibited superior residual activity compared to CR3. Although both oils achieved 100% repellency after 24 h at 25% concentration, CR9 maintained higher repellency over time, with a mean repellency index of 64 ± 4% after 28 days versus 25 ± 3% for CR3. Additionally, CR3 became significantly less repellent than CR9 after 21 days. These findings suggest that CR9 provides a stronger baseline for developing an enhanced formulation. By combining CR9 oil with citronellol and carvacrol, our goal was to leverage its inherent potency and extend its effective duration beyond what catnip oil alone can achieve.

Higher application rates are commonly used in human-skin repellency assays because the skin absorbs and volatilizes repellent compounds, and because strong host cues such as heat, odor, and moisture must be overcome for repellency to be detected. This pattern is reflected in regulatory guidelines, including the U.S. EPA OPPTS 810.3700 guideline for insect repellents applied to human skin [38] and the WHO protocol for skin-applied repellents [39], both of which specify relatively high application rates for human-skin testing. Similarly, studies evaluating host-associated repellency have noted that stronger chemical signals are required to elicit measurable avoidance under conditions where host cues are present [40]. In accordance with these established practices, we used a higher dose (2.54 mg cm^−2^) in the finger-stimulus assay to ensure that repellency could be detected under strong host-seeking conditions. Future studies should incorporate a broader range of doses to develop a dose–response relationship and to identify the minimum effective concentration under both laboratory and field-relevant conditions. Another potential concern in the finger stimulus assay is whether bed bugs could have deposited fecal or aggregation cues on the textile surface. Bed bugs defecate only after consuming a blood meal, beginning immediately after feeding and continuing for approximately 7–11 d thereafter [41]. The insects used in this assay were 14–24 d unfed, which is well beyond the documented defecation period, and each individual contacted the textile surface for no more than 5 min without resting or aggregating. No fecal spots were observed during any trial. Under these conditions, deposition of fecal or aggregation cues is highly unlikely, and reuse of the Petri dishes did not introduce detectable bias into the repellency measurements.

Although 20% catnip CCC oil provided repellency for up to three days in semi-field arenas and up to six days in the finger-stimulus test, efficacy in infested apartments was limited to one day. Several environmental factors likely contributed to this discrepancy. First, the presence of a human host during most times of the day stimulates bed bug host-seeking behavior and makes them more likely to pass a repellent barrier. Second, essential oil components such as nepetalactones, citronellol, and carvacrol are highly volatile and degrade more rapidly under fluctuating temperature and humidity conditions typically found in occupied apartments, which increases evaporation rates and reduces residual activity [25,30]. Third, the continuous movement of bed bugs across treated surfaces in naturally infested apartments may also contribute to faster depletion or mechanical removal of residues. These combined environmental and substrate-related factors likely explain the shorter field efficacy observed. This outcome underscores the importance of evaluating repellents in naturally infested environments and highlights the influence of application parameters. The tested barrier was only 2.0 cm wide; broader barriers or higher concentrations may improve performance and warrant further investigation. For travelers or visitors who remain in infested locations for short periods, near-complete repellency is desirable. Future work should explore optimized barrier dimensions and slow-release formulations to extend efficacy.

The performance of the 20% catnip CCC oil formulation is influenced not only by its active terpenoid components but also by the excipients incorporated to enhance stability and delivery. PEG-200, a low-molecular-weight polyethylene glycol broadly used in topical and cosmetic products, functions as a solubilizer, co-solvent, and humectant, and can improve miscibility and moderate evaporation of volatile essential oil constituents [42,43]. Its presence in the formulation likely facilitated uniform dispersion of hydrophobic compounds such as nepetalactone, carvacrol, and citronellol, thereby supporting a more sustained release profile. In contrast, BHT acts as a chain-breaking antioxidant that protects monoterpenes and other labile constituents from oxidative degradation [44,45]. By limiting oxidation during storage and handling, BHT helps maintain chemical integrity and may contribute indirectly to the sustained repellency observed in bioassays. Together, these excipients enhance both the physicochemical stability and functional performance of the CCC oil formulation.

A practical limitation of catnip CCC oil is its strong odor, which may affect user acceptance. During field trials, only one resident commented on odor intensity, and participation continued; however, systematic surveys are needed to assess acceptability in real-world contexts. Another consideration is that catnip oil can elicit attraction in some domestic cats; users should avoid placing treated fabrics in areas accessible to pets, and future work should evaluate feline behavioral responses to the CCC formulation. Additionally, repellents should not be considered standalone solutions for bed bug management. Their role is complementary within integrated pest management programs, providing temporary protection in specific scenarios such as inspections or short-term stays. Overall, this study validates the effectiveness of a novel catnip-based formulation for fabric applications and identifies key factors influencing its performance. Further research should address formulation improvements, odor mitigation, and integration strategies with established control measures to enhance practical utility.

## 5. Conclusions

Twenty percent catnip CCC oil demonstrated strong short-term repellency against bed bugs across simulated field tests, human-stimulus assays, and naturally infested apartments. Its performance was comparable to DEET for up to three days under controlled conditions and remained effective for up to six days in the finger-stimulus test, though field efficacy lasted only one day. These results highlight the potential of catnip-based formulations as complementary, fabric-applied repellents for short-term protection, while emphasizing the need for improved barrier designs and slow-release technologies to enhance durability in real-world settings.

## Figures and Tables

**Table 1:** Chemical composition of the essential oil of catnip (*Nepeta cataria* cultivar CR9) obtained by GC-MS.

Peak	RI *	RT *	Compound	Percent of Peak Area *
1	917	7.544	*α*-thujene	T
2	926	7.643	*α*-pinene	3.0 ± 3.0
3	1015	8.648	*p*-cymene	T
4	1193	10.229	Unidentified 1	1.2 ± 0.4
5	1382	11.632	(*Z*, *E*)-nepetalactone	79.8 ± 6.9
6	1414	11.851	(*E*, *Z*)-nepetalactone	3.5 ± 1.2
7	1425	11.921	*β*-caryophyllene	5.3 ± 1.8
8	1461	12.158	*α*-humulene	T
9	1535	12.638	Hexyl benzoate	T
10	1591	12.983	Caryophyllene oxide	5.9 ± 2.5
11	1644	13.296	Unidentified 2	T
			Total Identified Peaks	98.7
			Total Unidentified Peaks	1.3

* The relative peak percentage area of the compounds is presented as the mean of three replicates ± Standard Deviation. RI—Kovats Retention Index generated from a series of n-alkanes (C8–C18); RT—Retention Time (min). T—Trace amount (less than 0.5%).

## Data Availability

The raw data supporting the conclusions of this article will be made available by the authors on request.
